# Supplementary material for: Identification of Freezing-Responsive microRNAs and Their Targets in Chinese Jujube by Small RNA and Degradome Sequencing
Source: Int J Mol Sci. 2026 Sep 4;27(17):7912. doi: 10.3390/ijms27177912 (PMC13566890; doi:10.3390/ijms27177912)
Supplement: Supplementary file 1 [file ijms-27-07912-s001.zip › Supplementary Material S2/novel_miR_25.pdf]

Provisional ID	:	novel_miR_25
Score total	:	1.6
Score for star read(s)	:	-1.3
Score for read counts	:	0
Score for mfe	:	1.3
Score for randfold	:	1.6
Score for cons. seed	:	
Total read count	:	116
Mature read count	:	59
Loop read count	:	57
Star read count	:	0

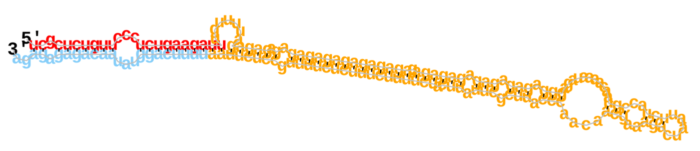

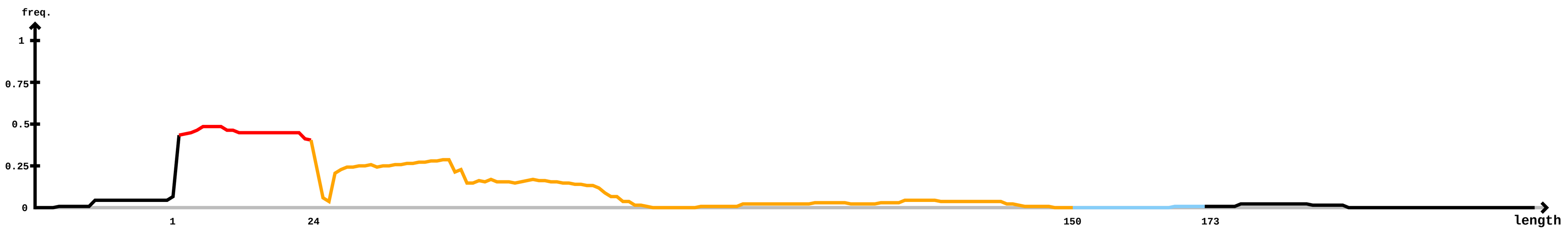

Mature

Star

5'- cucuuuucucucucugcaacguucgcucuguucccucugaagauuuguuuuagagagagagagagagagagagagagagagagagagagagagggagauaaaacaaugccaucuuaaucagaaagcaacaacccauucgcuuauucaucuuuucuuucucuuuugccucuuaauuuucagguauaacagagagagagagaaaagagagggaacagaggaauauauauaauacauuucuuuuuuuaauucauga -3'

((((.((((((((((....(.(((.(((((((...((((((((((.......(((((.(.((((((((((((((.((((((.(((.(((.(((..........(((..(((.....)))..)))....))).))).))).))).)))))))))))))))))).))))))))))))))))...))))))).))).)...)))))))))).)))).............................

exp	reads	mm	sample
cuuuucuUucucugcaacg	1	1	LT-3
ucucucugcaacguucgcucug	2	0	JCK-2
ucucucugcaacguucgcucug	1	0	JCK-1
ucucucugcaacguucgcucuguu	1	0	JCK-3
ucucucugcaacguucgcucuguu	1	0	JCK-2
uucgcucuguucccucugaaga	1	0	LT-1
uucgcucuguucccucugaaga	3	0	LT-3
ucgcucuguucccucugaagau	1	0	LT-3
ucgcucuguucccucugaagauu	1	0	LT-2
ucgcucuguucccucugaagauu	22	0	LCK-1
ucgcucuguucccucugaagauu	26	0	LCK-2
cgcucuguucccucugaagauu	1	0	JCK-3
gcucuguucccucugaaga	1	0	LT-3
cucuguucccucugaagauuuguuuuagag	2	0	LT-2
ucuguucccucugaagauuug	3	0	LT-2
Auuuuagagagagagagagagaga	3	1	LCK-3
uuuagCgagagagagagaga	14	1	JT-1
uuuagagagagagCgagagaga	3	1	JT-3
uuuagagagagagCgagagaga	6	1	JT-2
uuagagagagagagagagagagagaA	1	1	JCK-2
uuagaUagagagagagagagagaga	2	1	LT-3
uagagagagagagagagagagagGg	1	1	JCK-2
uagagagagagagagagagagagGg	1	1	LT-3
gagagagagagNgagagagagaga	8	1	JCK-1
gagagagagagNgagagagagaga	8	1	JCK-1
gagagagagagNgagagagagaga	8	1	JCK-1
gagagagagagNgagagagagaga	8	1	JCK-1
gagagagagagNgagagagagaga	8	1	JCK-1
gagagagagagNgagagagagaga	8	1	JCK-1
gagagagagagNgagagagagaga	8	1	JCK-1
gagagagagagNgagagagagaga	8	1	JCK-1
Uagagagagagagagagagagagaggg	1	1	JCK-2
Uagagagagagagagagagagagaggg	1	1	LT-3
gagagaCagagagagagagagagaggga	2	1	LT-3

Mature **Star**

cucuuuucucucucugcaacguucgcucuguucccucugaagauuuguuuuagagagagagagagagagagagagagagagagagagggagauaaaacaaugccaucuuaaucagaaagcaacaacccauucgcuuauucaucuuuucuuucucuuuugccucuuaauuuucagguauaacagagagagagaaaagagagggaacagaggaauauauauaauacauuuucuuuuuuuaauucauga

...agagagagagagaAagagagaggg...	2	1	JCK-2
...gagaCagagagagagagagaggga...	1	1	JCK-3
...gaAagagagagagagagagagg...	2	1	LT-3
...gagagagaAagagagagggaga...	3	1	LT-2
...gagagagagagagagagagggaCa...	1	1	LT-3
...gagagCgagagagagggagaua...	1	1	LT-3
...agagagagagagggagauaaaa...	1	0	LT-1
...gagagagagagggagauU...	2	1	LT-3
...agagagagagggagauaaa...	1	0	LT-3
...ucuuaaucagaaagcaacaacccau...	1	0	LT-3
...cagaaagcaacaacccauucgc...	2	0	LT-3
...acccauucgcuuauucaucuu...	1	0	LT-1
...uuauucaucuuuucuuucucuu...	2	0	LT-2
...uauucaucuuuucuuucucuuuugccucu...	1	0	JCK-2
...caucuuuucuuucucuuuu...	1	0	LT-3
...caucuuuucuuucucuuuug...	1	0	LT-1
...gagagagaaaagagagggaacag...	1	0	LT-1
...gagagggaacagaggaau...	2	0	LT-3
